# The moderating effect of cognitive impairment on the relationship between inner speech and auditory verbal hallucinations among chronic patients with schizophrenia

**DOI:** 10.1186/s12888-023-04940-4

**Published:** 2023-06-14

**Authors:** Daniella Mahfoud, Souheil Hallit, Chadia Haddad, Feten Fekih-Romdhane, Georges Haddad

**Affiliations:** 1 Faculty of Science, Lebanese University, Fanar, Lebanon; 2 School of Medicine and Medical Sciences, Holy Spirit University of Kaslik, P.O. Box 446, Jounieh, Lebanon; 3 Applied Science Research Center, Applied Science Private University, Amman, Jordan; 4 Research Department, Psychiatric Hospital of the Cross, Jal Eddib, Lebanon; 5 INSPECT-LB (Institut National de Santé Publique, d’Épidémiologie Clinique Et de Toxicologie-Liban), Beirut, Lebanon; 6 School of Health Sciences, Modern University for Business and Science, Beirut, Lebanon; 7 The Tunisian Center of Early Intervention in Psychosis, Department of Psychiatry “Ibn Omrane”, Razi Hospital, 2010 Manouba, Tunisia; 8 Faculty of Medicine of Tunis, Tunis El Manar University, Tunis, Tunisia

**Keywords:** Inner speech, Schizophrenia, Positive symptoms, Auditory verbal hallucinations, Cognitive impairment

## Abstract

**Background:**

Even though there is an increasing amount of evidence from behavioral and neuroimaging studies to suggest that pathological inner speech plays a role in the emergence of auditory verbal hallucinations (AVH), studies investigating the mechanisms underlying this relationship are rather scarce. Examining moderators might inform the development of new treatment options for AVH. We sought to extend the existing knowledge by testing the moderating role of cognitive impairment in the association between inner speech and hallucinations in a sample of Lebanese patients with schizophrenia.

**Methods:**

A cross-sectional study was conducted from May till August 2022, enrolling 189 chronic patients.

**Results:**

Moderation analysis revealed that, after controlling for delusions, the interaction of experiencing voices of other people in inner speech by cognitive performance was significantly associated with AVH. In people having low (Beta = 0.69; t = 5.048; *p* < .001) and moderate (Beta = 0.45; t = 4.096; *p* < .001) cognitive performance, the presence of voices of other people in inner speech was significantly associated with more hallucinations. This association was not significant in patients with high cognitive function (Beta = 0.21; t = 1.417; *p* = .158).

**Conclusion:**

This preliminarily study suggests that interventions aiming at improving cognitive performance may also have a beneficial effect in reducing hallucinations in schizophrenia.

## Background

Even though schizophrenia has been recognized as a disease for over a century, its etiology and pathophysiology remain uncertain [1]. According to the Diagnostic and Statistical Manual of mental disorders DSM-5, schizophrenia is characterized by abnormalities in one or more of the following five domains: hallucinations, delusions, disorganized thinking or speech, grossly disorganized or abnormal motor behavior, and negative symptoms [2]. Positive symptoms are the quickest to detect, and the reason behind the term “positive” is that the abnormality resides in their additional presence [3]. Positive symptoms include delusions and hallucinations which are cornerstones of psychosis; they are prominent because they are severe, frequent, and are the primary target of schizophrenia pharmacotherapies [4]. Hallucinations can happen in every sensory modality, although auditory verbal hallucinations (AVH) are the most prevalent [5], affecting two thirds of people diagnosed with schizophrenia [6]. AVH refer to subjective perceptual experiences of voices occurring in the absence of external stimuli. Because the voices are characterized by a negative emotional content (e.g., criticize/threaten the patient or command them to behave against their desires), AVH may be quite distressing [7]. In addition, hallucinations may negatively affect interpersonal functioning and even lead to dangerous behaviors [8]. Besides, AVH are sometimes associated with drug resistance in patients with schizophrenia [9]. Despite this, the neural and cognitive mechanisms underlying AVHs require more investigation. Therefore, it is important to understand how AVH emerge to be able to enhance the effective, theoretically-based psychological interventions (e.g. CBT for psychosis) [10]. In this regard, we focus in the present work on the pathway leading from inner speech to AVH.

### The relationship between inner speech and AVH

Inner speech is the egocentric use of language while omitting overt and perceptual articulation [11]. It has been suggested that AVH may emerge from inner speech misattribution to external (non-self) sources [12, 13]. This could be caused by schizophrenia patients' defective auditory cortical reactivity to inner speech, and may lead to a misidentification of inner speech as external voices [14]. The auditory pathway's activation determines how internal and external speech are localized in space. Therefore, malfunction of this circuit may lead to an outer spatial perception of the inner voice which can explain the outside-the-head interpretation of the inner voice that arises in AVH [15], even though some voice-hearers perceive their voices as occurring inside their head which may be regarded as a form of inner speech [16]. Patients with schizophrenia experiencing delusions have also been shown to have challenges in self-monitoring their speech [17] and are as well prone to experience external misattributions [18]. Hence the importance of adjusting for delusions when examining the relationship between inner speech and AVH.

Previous research revealed a relationship between particular inner speech features and an increased tendency for auditory hallucinations. The dialogic component and other people in inner speech related more strongly to auditory hallucination tendency than other factors [19]. Besides, individuals with psychosis experienced high levels of evaluative inner speech, which also positively correlated with the severity of AVH [20]. Even though there is an increasing amount of evidence from behavioral and neuroimaging studies to suggest that pathological inner speech plays a role in the emergence of AVH [21–23], studies investigating the mediators/moderators underlying this relationship are rather scarce. In order to further understand the interrelations between inner speech and AVH, we examined the moderating effect of an important construct that has been related to the development and experience of AVH, cognitive impairment [24, 25]. A moderator is a variable that affects either the direction or the strength of the association between an independent variable (here, inner speech) and an outcome variable (AVH, in this study) [26]. Examining moderators is thus necessary as it may impact the association between inner speech and the development of AVH and might as well inform the development of new treatment options for AVH.

### Cognitive impairment as moderator in the relationship between inner speech and AVH

Cognitive impairment seems to be a possible factor moderating the cross-sectional relationship from inner speech to AVH, being especially linked to both variables. Impaired cognitive performance seems to be a central characteristic of schizophrenia, with 70–80% of patients presenting cognitive impairments in the form of significant general intellectual impairment and executive functioning problems [27]. Previous research has demonstrated that cognitive deficits may predispose people to psychiatric disorders, could serve as an early indicator of future illness, and predict the disease prognosis [28]. More particularly, a variety of hallucinatory cognitive mechanisms such as verbal memory and language processing have been hypothesized [29]. It is proposed that hallucinations are caused by a failure in self-recognition due to deficiencies in source-monitoring mechanisms, which lead mental events to be misattributed to external sources [25]. These often involve processes that assess memory recordings in order to form a concise overview of an event [30]. Therefore, cognitive impairment, including working memory deficits, may predict AVHs and could contribute to their emergence [24].

On the other hand, there is evidence that inner speech is closely linked to cognitive function. Inner speech supports executive functioning related to task switching performance [31], and is considered to play some role in mediating short-term/working memory [32]. Besides, inner speech disturbances may affect socio-emotional functioning, and general cognitive processing such as silent remembering, reading, writing [33], planning, behavioral control, inhibition, cognitive flexibility [11], and attentional improvement [34]. Cognitive impairment can affect the underlying cognitive processes normally involved in inner speech self-monitoring [17], leading to uncontrolled inner speech that can result in inner speech-based AVH [35]. It is also proposed that certain executive function deficiencies are what leads to unregulated inner speech, and when inner speech is uncontrolled, as it is frequently used in executive function activities, it will result in additional cognitive issues [36].

### The present study

While a deficit in inner speech has been one of the most popular cognitive theories about the cause of AVH [25], there is a lack of evidence on the nature of this association. Therefore, it becomes evident that further investigating pathways between inner speech and AVH is strongly needed. To our knowledge, the only studied pattern was the mediating effect of dissociation between varieties of inner speech (other people and evaluative) and hallucination proneness [37]. Furthermore, although inner speech is a commonly experienced phenomenon in daily life, it is still poorly understood. People perceive inner speech regardless of their language and culture [33]; however speech cannot exist outside the framework of interpersonal and sociocultural interactions. The core evolution of the human being occurs in a social context, moving from the social to the individual [38]. Besides, culture influences a person with voice-hearing experiences and clinical repercussions might result from these variations [39]. Hence the importance of this study which is the first to research inner speech in an Arabic-speaking population from a Middle East country, Lebanon. In the present study, we sought to extend the existing knowledge by testing the moderating effect of cognitive impairment in the association between inner speech and hallucinations in Lebanese patients with schizophrenia. We propose that cognitive impairment enhances the cross-sectional positive correlation between particular inner speech types and AVH in these patients.

## Methods

### Study design and participants

**Fig. 1:**
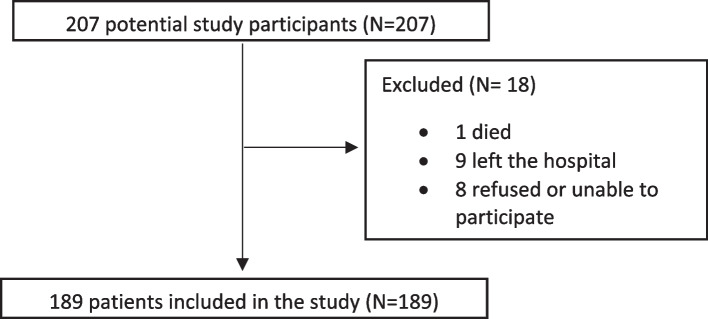
Flow chart of enrollment of patients in the study

A cross-sectional study was conducted between May and August 2022, at the Psychiatric Hospital of the Cross, Lebanon, recruiting 207 randomly selected in-patients diagnosed with schizophrenia. Patients who were aged 18 or older, cognitively able to fill a questionnaire, and classified as having chronic schizophrenia according to the Diagnostic and Statistical Manual of Mental Disorder, Fifth Edition (American Psychiatric Association, 2013) were eligible to participate. Patients with any other diagnosis, and those who refused to participate were excluded from the study. As a result, 18 of the 207 individuals recruited were eliminated, leaving 189 patients to compose our sample (Fig. 1). Data was collected through performing personal face-to-face structured interviews with the participating subjects. Interviews were conducted after obtaining the signed consent form. Only participants who were able to provide consent were included in the study. Those who were unable to do so (8 participants) due to severe cognitive impairment or psychopathology were excluded to not affect the reliability or validity of our assessments.

### Measures

The questionnaire was in Arabic, the native language of the country, and took around 20 min to complete. The first section was filled out using the patients’ medical files, and it assessed the socio-demographic and clinical characteristics of the patients. These include age, sex, marital status, education level, income, duration of hospitalization, duration of schizophrenia illness and family history of psychiatric illness, along with the socio-economic status of the patients, which was categorized as low (< 200 USD), intermediate (200–500 USD), or high (> 500 USD) based on the patient's caregivers’ monthly income.

The following scales were included in the second section of the questionnaire, which was completed during individual interviews with the patients.

### Varieties of Inner Speech Questionnaire – Revised (VISQ-R)

The Varieties of Inner Speech Questionnaire – Revised (VISQ-R) was employed to assess individual variations in the phenomenological qualities and characteristics that distinguish the participants' usual inner speech [13, 19]. The VISQ-R includes 26 items rated on a 7-point Likert scale (1 = Never, 7 = All the time). It includes the following five subscales: Dialogic inner speech, i.e. inner speaking that is conversational (5 items), Condensed inner speech, i.e. inner speaking that is shortened while retaining its meaning (5 items), other people in inner speech, i.e. experiencing other’s imagined voices when inner speaking (5 items), evaluative/ critical inner speech, i.e. assessment of action that is critical and frequently negative using inner speech (7 items), and positive/regulatory inner speech, i.e. positive and constructive inner speaking (4 items) [19]. The Cronbach’s alpha values were as follows: condensed (α = 0.96), dialogic (α = 0.96), evaluative (α = 0.85), other people (α = 0.89), positive (α = 0.93).

### Psychotic Symptom Rating Scales (PSYRATS)

The PSYRATS [40] is a multidimensional assessment of delusions and auditory hallucinations with 17 items and a five-point Likert scale (0–4). The items for auditory hallucinations (11 items) are: frequency, duration, location, loudness, beliefs about origin of the voices, amount and intensity of negative content, amount and intensity of distress, disruption of life and controllability. The items for delusions (6 items) are: amount and duration of preoccupation, conviction, amount and intensity of distress and disruption of life caused by beliefs. Each subscale score is determined by adding together the scores of its items, with higher scores indicating more severe symptoms. The AVH and delusions subscales had internal reliability coefficients of 0.96 and 0.94, respectively.

### Montreal Cognitive Assessment (MoCA)

Montreal Cognitive Assessment or MoCA Test [41] is a widely used screening assessment tool for detecting mild cognitive impairment (MCI). MoCA has been implemented in clinical settings and is widely used as a scale in research. It has eight sections covering the following cognitive domains: Visuospatial abilities, executive functions, attention, memory, concentration, language, verbal abstraction, and orientation. As such, the MoCA covers a wider range of cognitive functions than other cognitive instruments [42–44], and provides information about general cognitive functioning disturbances in patients with schizophrenia, the MoCA test; and for schizophrenia patients. Our results suggest that the MoCA test is a useful screening instrument for assessing cognitive impairment in psychotic patients and has some advantages over other available instruments, such as its ease-of-use and short administration time. The MoCA has demonstrated its utility in schizophrenia patients aged over 18 years [42, 45]. A MoCA cutoff score of 23 allows for differentiating healthy cognitive aging from possible Mild Cognitive Impairment [46]. The Arabic translated version of the MoCA test is validated in Lebanon as an effective instrument for assessing cognitive deficits in schizophrenia [47]. The test had an internal reliability coefficient of 0.81.

### Translation procedure of the scales

The original English versions of the VISQ-R and the PSYRATS were independently translated by two mental health professionals. The first one translated from English to Arabic. The Arabic version of the scales was then blindly back translated into English by the second professional. The two English versions were compared in order to discern discrepancies and solve any inconsistencies between the two versions until full agreement was found.

### Statistical analysis

All data was analyzed using SPSS version 26. Cronbach alphas were calculated for the assessed subscales to gain insight into their internal consistency. The student’s independent t-test was used to compare continuous variables in two groups. Relationships between the continuous variables were first analyzed using Pearson’s test. The PROCESS v.3.4 model 1 was used to conduct the moderation analysis; results were adjusted over all variables that showed a p < 0.25 in the bivariate analysis. A p < 0.05 was considered significant.

## Results

### Sample characteristics

A total of 189 patients enrolled in this study. Their mean age was 57.26 ± 11.20 and 61.9% males. The majority was from a low socioeconomic status (54.7%) and single (84.7%). More details about the sample can be found in Table 1.

**Table 1:** Sociodemographic and other characteristics of the patients (*N* = 189)

Variable	N (%)
Gender	
Male	117 (61.9%)
Female	72 (38.1%)
Education	
Illiterate / Primary	76 (40.2%)
Complementary	60 (31.9%)
Secondary	30 (16.0%)
University	22 (11.7%)
Socioeconomic status	
Low	160 (84.7%)
Intermediate	50 (26.6%)
High	3 (1.6%)
Marital status	
Single	160 (84.7%)
Married	10 (5.2%)
Divorced	2 (1.1%)
Widowed	17 (9.0%)
Family history of psychiatric illness	
No	128 (67.7%)
Yes	61 (32.3%)
	**Mean ± SD**
Age (in years)	57.26 ± 11.20
Duration of illness (in years)	28.21 ± 13.61
Duration of hospitalization (in years)	14.07 ± 10.68
MoCA score	13.72 ± 6.33
VISQ condensed	23.64 ± 10.40
VISQ dialogic	19.04 ± 10.25
VISQ evaluative	25.90 ± 10.87
VISQ other people	14.02 ± 8.75
VISQ positive	19.96 ± 6.65
Hallucinations score	10.19 ± 13.28
Delusions score	9.48 ± 7.84

### Bivariate analysis

The results of the bivariate analysis can be found in Tables 2 and 3. Higher dialogic, evaluative and other people inner speech scores were significantly associated with greater hallucinations.

**Table 2:** Bivariate analysis of categorical variables associated with the hallucinations scores

Variable	Hallucinations (mean ± SD)	*p*
Gender		.890
Male	10.09 ± 13.12	
Female	10.36 ± 13.64	
Education		.068
Illiterate / Primary	12.70 ± 14.38	
Complementary	8.85 ± 12.58	
Secondary	10.87 ± 13.21	
University	4.73 ± 9.58	
Socioeconomic status		.233
Low	10.97 ± 13.50	
Intermediate / High	8.40 ± 12.71	

Numbers in bold indicate significant *p*-values

**Table 3:** Correlations of continuous variables associated with the hallucinations scores

Variable	1	2	3	4	5	6	7	8	9	10
1. Hallucinations	1									
2. Delusions	.26^***^	1								
3. Cognitive performance	-.12	-.15^*^	1							
4. Age	-.12	.14	-.22^**^	1						
5. Duration of illness	-.02	.08	-.24^**^	.62^***^	1					
6. Duration of hospitalization	.03	.08	-.21^**^	.44^***^	.53^***^	1				
7. VISQ condensed	.02	-.10	-.25^**^	-.06	.01	-.12	1			
8. VISQ dialogic	.57^***^	.25^**^	.06	-.10	.02	-.004	-.05	1		
9. VISQ evaluative	.36^***^	.25^**^	.03	-.10	.01	.12	-.16^*^	.43^***^	1	
10. VISQ positive	.10	-.16^*^	.16^*^	-.07	-.06	-.11	.04	.13	.23^**^	1
11. VISQ other people	.52^***^	.26^***^	.02	-.03	.01	.12	-.23^**^	.46^***^	.56^***^	.09

^*^ *p* < 0.05, ^**^ *p* < .01, ^***^ *p* < .001

VISQ-R: the revised Varieties of Inner Speech Questionnaire

### Moderation analysis

**Fig. 2:**
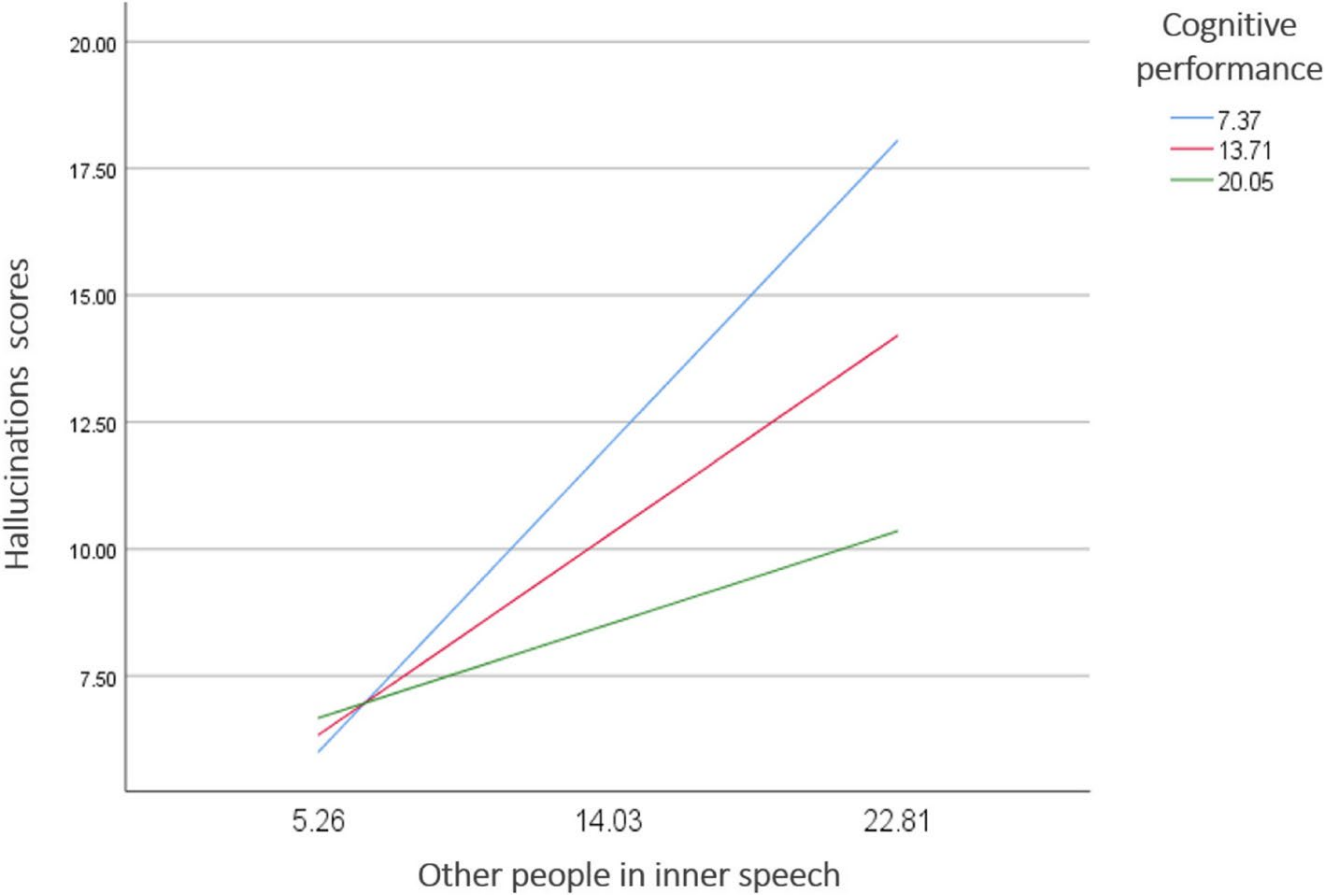
Association between the interaction other people in inner speech by cognitive performance and hallucinations

Higher other people in inner speech scores were significantly associated with more hallucinations (Beta = 0.96; t = 4.454; *p* < 0.001), whereas cognitive function was not associated with hallucinations (Beta = 0.25; t = 0.993; *p* = 0.322). After controlling for delusions, the interaction other people in inner speech by cognitive performance was significantly associated with hallucinations (Table 4). In people having low (Beta = 0.69; t = 5.048; *p* < 0.001) and moderate (Beta = 0.45; t = 4.096; *p* < 0.001) cognitive performance, the presence of other people in inner speech was significantly associated with more hallucinations (Fig. 2). This association was not significant in patients with high cognitive performance (Beta = 0.21; t = 1.417; *p* = 0.158).

**Table 4:** Moderation analysis: Association of the interaction between each inner speech subscale score by cognitive performance (MoCA scores) with hallucinations

	**Effect**	**SE**	**T**	***p***
VISQ condensed	.01	.01	.644	.520
VISQ dialogic	-.004	.01	-.315	.753
VISQ evaluative	-.01	.01	-.873	.384
VISQ other people	-.04	.01	-2.62	.010^*^
VISQ positive	-.11	.27	-.406	.685

^*^Indicates significant interaction. Results are adjusted over the following variables: the other four inner speech scores, level of education, socioeconomic status, age and delusions score. *MoCA* the Montreal Cognitive Assessment test, *VISQ-R* the revised Varieties of Inner Speech Questionnaire. Bold value: significant at *p* < 0.05

## Discussion

An examination of the moderating effect of cognitive impairment is of high relevance for understanding the unclear interplay between inner speech and AVH. This study revealed that, after controlling for delusions, cognitive performance had a moderating effect on the positive relationship between a specific type of inner speech, i.e. other people in inner speech, and hallucinations. To our knowledge, this investigation is by far the first to study the moderating role of cognitive impairment in the relationship between inner speech and AVH. In addition, to date, this study is the first in its nature that investigates a culturally-dependent concept, i.e. inner speech, among patients with schizophrenia in an Arabic-speaking country of the Middle East and North African region.

We found that among schizophrenia patients, evaluative/critical inner speech was the most expressed quality of inner speech followed by condensed, positive, dialogic, and other people in inner speech. The correlation analysis indicated that condensed inner speech correlated to worse cognitive performance, whereas positive inner speech was associated with higher cognitive performance. Condensed inner speech was shown to be related to psychopathology [48], and the inclination to engage in condensed inner speech is inversely associated to convergent thinking capacity [49]. On another note, positive/regulatory inner speech may have cognitive benefits in the domains of creativity and imagination [19], which might explain the positive association between positive inner speech and MoCA scores. Previous research found that dialogic and evaluative inner speech predicted cognitive disorganization [50], and are most likely to affect executive processes especially that a critical evaluation of one’s performance is likely to be disruptive [36]. However, no associations were discovered in this study. It is possible that the sample size or the specific measures used in this study were not sensitive enough to detect these associations, or that other factors not accounted for in this study may have influenced the results. Additionally, it is important to note that the relationship between inner speech and cognitive functioning is complex and may vary depending on the specific cognitive domain or task being assessed [51]. Therefore, it is possible that dialogic and evaluative inner speech may be more strongly related to cognitive functioning in certain contexts or for specific types of tasks that were not assessed in this study.

It was also demonstrated that evaluative, dialogic, and other people in inner speech correlated with AVH, which supports previous findings [20, 37, 48]. The relationship between dialogic inner speech and hallucinations is proposed to be bidirectional. It is likely that having more dialogic inner speech makes it easier to communicate with the voices. However, it is also possible that voices motivate people to engage in greater inner communication that is dialogic [20]. Besides, the correlation with evaluative inner speech may be due to the fact that the same factors affecting negative AVH may affect, in turn, ongoing inner speech, including adverse life experiences, emotion regulation approaches, the presence of physical or social threat, and a bad relationship with AVH [52]. In addition, positive inner speech has been shown to negatively correlate with delusions, as delusional content is most commonly persecutory and negative [53]. Since it is very common for AVH and delusions to coexist, and given that a unifying factor among these two is external agency, delusions should be controlled for in order to confirm the association between these aspects of inner speech and AVH. Moreover, higher other people in inner speech scores were significantly associated with more hallucinations, which is concordant with previous studies [20, 37, 48]. AVH are commonly perceived as social entities distinctive from self [54]. Furthermore, inner speech is thought to be the result of an internalization of linguistically mediated social interactions with other people, which, according to Vygotsky's model, are transformed into an internal dialogue with the self [55]. This internalization of the dialogue may not have been successful in individuals with schizophrenia. Therefore, the dominance of other people in inner speech in psychiatric patients experiencing AVH suggests that these voices may be misattributed to external sources.

After controlling for delusions, our moderation analysis has shown that in patients having low and moderate cognitive performance, higher other people in inner speech was significantly associated with more AVH, which was not the case in patients with high cognitive performance. It is interesting to note here that the majority of the participating patients (40.2%) are illiterate which might have affected their cognitive functioning and performance, especially that higher level of education is coupled with greater long-term enhancements in cognitive performance [56]. This preliminarily finding presumes that cognitive impairment may play a role in the attribution of these voices perceived inside the head to external sources. A recent study has found that verbal hallucination proneness in a non-clinical sample was associated with higher rates of false recognition of high-frequency words and activation of language and decisional brain areas during false recognition of low-frequency words, suggesting a failure in the self-monitoring of inner speech [57]. These findings are consistent with the idea that inner speech may play a role in the development of auditory verbal hallucinations and that individuals who are prone to hearing voices may have difficulty distinguishing between their internal thoughts and external stimuli. Many theories concerning the origin of AVH claimed that impaired cognition leads to reduced inhibitory control which is suggested to result in the emergence of hallucinations [58]. Indeed, cognitive factors have been proposed to dissociate imagery and perception; and determine whether the source of sensory signals is externally or internally generated. Following the source monitoring framework, higher-level cortical circuits would be responsible for differentiating between cognitively-produced internal events (or inner speech) and perceived speech [59]. In the hallucinating brain, abnormal abundant inner speech may reflect a bias towards external sources and an impairment in reality monitoring. In other words, internally generated representations appear as if they were easily produced without cognitive effort, making them mistaken for perception triggered by external sources [60]. Several other cognitive functions like self-monitoring [35], and speech processing, the inhibition of irrelevant verbal information, have been shown to be impaired in patients experiencing AVH [61].

Consequently, there is a need to focus on managing these specific elements of cognitive performance in order to help patients better monitor their inner speech and prevent the misattribution of the presence of other people's voices in inner speech to external sources, perceived as AVH. Thus, cognition and cognitive mechanisms underlying AVHs may be caused by a deficit in monitoring of inner speech; and cognitive impairment may, in turn, lead to misattributing the voices of others perceived in the inner speech as external voices experienced as AVH. Whereas a better monitoring of inner speech leads to a better cognitive performance which entails better controllability of the voices.

### Clinical implications and research perspectives

Our findings could help in better understanding the interplay between each type of inner speech and AVH in patients with schizophrenia. This may help inform the development of tailored preventions and interventions in order to ameliorate the inner experience and the disease prognosis. Many therapies focus on the patient’s internal monologue such as compassionate focused therapy (CFT), which aims to establish more compassionate inner speech by changing content from negative or persecutory to more positive and comforting [62]. Cognitive behavioral therapy may also be effective since a number of cognitive mechanisms are likely to play a role in the development of inner speech based-AVH [63]. Psychoeducation can help in explaining how inner speech arises and the many ways in which people might perceive it, such as varieties of inner speech that are perceived as other people's voices, and how stress can make it challenging to identify thought patterns as self-generated [63].

One important finding of this study that has great implication for clinical outcomes, future development in treatment, and for research, is that cognitive impairment moderates the cross-sectional, positive relationship between other people in inner speech and AVH. In other words, cognitive impairment has a strengthening effect on this relationship. This means that interventions aiming at improving cognitive performance (e.g., cognitive remediation [64]) may also have a beneficial effect in reducing hallucinations. We recommend further attention on inner speech as a possible causal factor of AVH, and call for more cross-cultural research on the direct relationship inner speech-hallucinations, and its potential moderators.

Future research can focus on other potential moderators of this association, such as insight, since lack of insight, or unawareness of one's mental disease is a common occurrence in psychiatric disorders [65], and insight has been suggested to rely on metacognitive skills for self-reflection and perspective-taking [66]. Moreover, individuals become self-aware of their current mental state when they engage in higher order cognition through self-talk about states of mind and personality traits [67]. Therefore, it is essential to explore other potential moderators related to inner speech and schizophrenia symptoms to improve the management of the condition.

## Limitations

This study presents some limitations that need to be addressed in future research. Since it is a cross-sectional study, it cannot directly address the causal or mechanistic role of inner speech in the development of schizophrenia symptoms or cognitive disturbances. As such, longitudinal studies are required to confirm our assumptions. In addition, we may have a reporting bias since the main source of information is the patients describing their subjective inner speech experience and participant–researcher interaction bias since it is not self-filled and it may not have reflected participants’ actual experiences of inner speech. This methodology relies heavily on the patient’s metacognitive ability to reflect about thoughts, and this ability may be affected in some patients [68]. One strategy to overcome the constraints of the VISQ-R in future research is to reproduce our findings using more ecologically valid ways of evaluating inner speech, such as forms of experience sampling [11]. This method allows for inner experience to be captured in the moment. In addition, we did not collect and control for pharmacological data (e.g., type of antipsychotic drug, routes of administration, dose, duration of therapy, monotherapy/ combination), which might have affected our findings. Future studies should consider adjusting for these variables to rule out potential for residual confounding. Besides, our study only included patients from a single psychiatric hospital. This may have limited the sample size and the generalizability of our findings to other populations or clinical settings. Future studies should aim to recruit patients from multiple sites to enhance the validity of the results. Finally, we included chronic patients with schizophrenia who have been exposed to long-term effects of psychotic symptoms, antipsychotic therapy, and cognitive impairment; all these factors may interfere with our results. We thus recommend reproducing this study in patients with early stages of the disease.

## Conclusion

This study extends and advances the existing research by showing that cognitive impairment tended to strengthen the association between other people in inner speech and AVH severity. These findings, although exploratory and require additional investigation, can provide a foundation for a better understanding of schizophrenia positive symptoms and the patients' inner experiences, and how cognitive performance may affect the association between these two. Despite its limitations, we believe that this study provides novel insight into the relationships between the varieties of inner speech, AVH, and cognition, and embodies an early step toward fruitful hypotheses for future research to address.

## Data Availability

The datasets generated and/or analysed during the current study are not publicly available due to restrictions from the ethics committee (third party data owner) but are available from the corresponding author (S.H.) on reasonable request.
